# Bladder Preservation in Muscle-Invasive Bladder Cancer: A Population-Based Analysis from British Columbia

**DOI:** 10.3390/curroncol32120699

**Published:** 2025-12-11

**Authors:** Guliz Ozgun, Abraham Alexander, Gregory Arbour, Christian Kollmannsberger, Bernhard J. Eigl, Sunil Parimi

**Affiliations:** 1 BC Cancer Vancouver Center, Medical Oncology, Vancouver, BC V5Z 4E6, Canada; ckollmannsberger@bccancer.bc.ca (C.K.); bernie.eigl@bccancer.bc.ca (B.J.E.); 2 BC Cancer Victoria Center, Radiation Oncology, Victoria, BC V8R 6V5, Canada; aalexander3@bccancer.bc.ca; 3 Data Science Institute, University of British Columbia, Vancouver, BC V5Z 4E6, Canada; greg.arbour@ubc.ca; 4 BC Cancer Victoria Center, Medical Oncology, Victoria, BC V8R 6V5, Canada

**Keywords:** bladder preservation, radiation, chemoradiation, personalized medicine, MIBC

## Abstract

Bladder cancer is the 5th most common cancer in Canada and a quarter of diagnosed patients have muscle-invasive bladder cancer (MIBC). Standard treatment options, systemic therapy and radical cystectomy (RC) are associated with high rates of adverse outcomes. Recently, trimodal treatment (TMT), a bladder preservation strategy defined as maximal transurethral resection of bladder tumor (TURBT) and chemoradiation, has been considered an alternative per guidelines for select patients who prefer bladder preservation or those with comorbidities. Nevertheless, the uptake of bladder preservation strategies in Canada remains low. We conducted a retrospective evaluation in British Columbia (BC) to assess the real-world outcomes of bladder-sparing radiotherapy. Cohort treated with combined chemoradiotherapy (concurrent and/or adjuvant, neoadjuvant chemotherapy) showed numerical improvements across all evaluated endpoints, including disease-specific survival and progression-free survival, compared with radiation therapy alone, which is generally considered an inferior strategy. However, these differences did not reach statistical significance, contrasting with the literature. Despite the limitations posed by the small sample size and the study’s retrospective design, the findings highlight the pivotal role of appropriate patient selection in achieving meaningful therapeutic outcomes. Future studies integrating biomarker-driven strategies are needed to enhance outcomes through individualized treatment selection, particularly for older patients with multiple comorbidities.

## Simple Summary

This study examined bladder-preserving treatment strategies in an older patient population with multiple comorbidities who may not be ideal candidates for surgery. Despite the small sample size and retrospective design, the results showed that radiotherapy alone achieved outcomes similar to those of chemoradiation for some patients. Since chemoradiation has traditionally been considered the standard bladder-preserving approach, these findings suggest that, with careful patient selection, certain individuals may achieve comparable disease control with radiotherapy alone. This underscores the importance of individualized treatment planning in this vulnerable population. Future research should focus on integrating biomarker-driven and personalized strategies to better identify which patients are most likely to benefit from each treatment approach, improving clinical outcomes and quality of life while minimizing unnecessary treatment risks.

## 1. Introduction

Bladder cancer is the 5th most common type of cancer in Canada, and the expected bladder cancer diagnosis was more than 12,000 cases in 2024, according to the Canadian Cancer Statistics [1]. At diagnosis, nearly 25% of patients present with MIBC, which typically requires definitive treatment approaches. The primary treatment options for MIBC include systemic treatment and RC or bladder-sparing approaches (radical radiotherapy +/− radiosensitizing chemotherapy). The RC approach, although effective, is associated with a 90-day complication rate of 60–80% and can result in decreased quality of life [2,3,4].

In response to those challenges, bladder sparing strategies such as TMT, defined as maximal TURBT and chemoradiation, have emerged as an alternative treatment option for select patients. It is endorsed as an option (alongside radical cystectomy) by the current guidelines [5,6]. TMT is typically offered to patients who prefer bladder preservation or have significant comorbidities precluding RC. Ideal candidates for TMT generally have low-volume solitary tumors amenable to complete TURBT, in the absence of multifocal or extensive CIS, significant hydronephrosis, or lymph node metastasis in patients with a functioning bladder [7]. The TMT approach allows for a more personalized treatment strategy that aims to preserve bladder function and offer a better quality of life while still effectively addressing cancer [8].

The efficacy of TMT has been demonstrated through various single-arm Phase 2 and 3 clinical trials, as well as propensity score-matched data from multiple institutions [9,10,11]. Although available data support TMT as a bladder-sparing treatment for MIBC in appropriately selected patients, with outcomes comparable to cystectomy, uptake in Canada has been low. A retrospective evaluation in British Columbia, Canada, was conducted to assess real-world outcomes in patients with MIBC undergoing bladder-sparing radiotherapy; radiotherapy (RT) alone versus chemoradiotherapy (chemo + RT).

## 2. Methods

The study was approved by the University of British Columbia Research Ethics Board (approval code: H23-00347-A001; approval date: 8 March 2023). Patients with MIBC were identified from the BC Cancer registry who received radiotherapy with curative intent, either with or without neoadjuvant (NAC), adjuvant, and/or concurrent chemotherapy between 1 January 2002, and 31 December 2020.

Patients with a pathologic diagnosis of MIBC treated with a curative intent radiation-based strategy were included. Baseline demographic information and laboratory investigations were ascertained during the initial consultation. The reason for choosing a bladder-preserving approach to MIBC treatment was reported. Radiation dose and fractionation schedule were documented. If therapy was not completed, the cause was ascertained from the chart. If chemotherapy was given concurrently with the radiation, the regimen used was provided. If chemotherapy was not given, the reason for its omission was determined. Salvage cystectomy was documented when applicable. The cause of death was obtained from the Discharge Abstract Database. Exclusion criteria include patients who did not have pathologic documentation of MIBC or those who underwent an up-front radical cystectomy.

## 3. Outcomes and Statistical Analysis

Descriptive statistics were employed to characterize the RT and chemo + RT groups. Overall survival (OS), disease-specific survival (DSS), and progression-free survival (PFS) were analyzed using the Kaplan–Meier method. Outcomes were compared between those receiving radical RT alone and those receiving chemo + RT. All analyses were performed using R statistical software (R version 4.2.0, R Foundation for Statistical Computing, Vienna, Austria). Statistical significance was defined as a two-tailed *p*-value of less than 0.05 for all tests. Because treatment was not randomly assigned, efforts were undertaken to balance the two treatment groups and adjust for possible confounding variables. We implemented overlap weights (OW), utilizing the entire dataset, and, because overlap weights are bounded between 0 and 1, they are resistant to extreme weights. With OWs, patients with extreme propensity scores (close to 0 or 1) are downweighted. In contrast, patients who could conceivably receive either treatment (propensity score close to 0.5) contribute the most to the estimate of the effect.

To generate propensity scores for the likelihood of receiving treatment, a logistic regression model was fit using the following seven predictor variables: age at diagnosis, pT, ECOG, carcinoma in situ, tumor-associated hydronephrosis, tumor focality, tumor size, and creatinine levels. Overfitting was avoided by generating propensity scores via 10-fold cross-validation, where the hold-out folds’ propensity scores were used to construct the overlap weights. The model performed well, with an out-of-fold accuracy of 80.2% (standard error = 2.4%) and AUC of 0.858 (standard error = 0.033). We also assessed the standard mean difference (SMD) of each covariate both before (Table 1) and after weighting (Table 2). SMD’s for the weighted sample ranged from −0.04 to 0.03, indicating a good balance between covariates after conditioning the OW. The overlap weights were then used to fit a weighted Cox model for each of the three endpoints with RT vs. Chemo + RT as the sole predictor variable. Confidence intervals (CIs) for regression coefficients in the weighted Cox models were estimated via bootstrap with 1000 repetitions. For each repetition, a bootstrap sample was generated by sampling with replacement from the original dataset, using the overlap weights as sampling probabilities. A model was fit on the bootstrap sample, and the regression coefficients were recorded. To estimate a 95% CI, we simply took the values at the 2.5th and 97.5th percentiles of the 1000 bootstrap coefficient estimates. Estimating CIs in this way is a viable alternative to more complex methods for a range of sample sizes and treatment prevalence scenarios [12]. Mode imputation was used to impute missing values for the CIS and ECOG variables, and median imputation was used to impute values for age at diagnosis and tumor size. Missingness for these four variables was assumed to be completely random, and the chosen imputation methods were selected based on their perceived clinical plausibility.

## 4. Results

Among 231 patients evaluated, 170 received RT and 61 received chemotherapy with RT (concurrent, neoadjuvant, adjuvant, or their combinations), defined as the chemo + RT group. Males dominated both groups, accounting for 74.5% of the total. The average age at the time of diagnosis was 78.5 years (SD: 9.39) and the RT group was older (mean age 81.2 years) than the chemo plus RT group (mean age 70.7 years). T2 stage disease was most common in both groups, with 72.4% (*n* = 123) in the RT group and 55.7% (*n* = 34) in the chemo + RT group. The T4 stage was the least common, representing 9.4% (*n* = 16) in the RT group and 16.4% (*n* = 10) in the chemo + RT group. CIS was present in 21.2% of patients (26.2% in chemo + RT vs. 19.4% in RT group), with tumor-associated hydronephrosis observed in 35.5% (41% in chemo + RT vs. 33.5% in the RT group) and multifocal tumors in 27.7% (29.5% in the chemo + RT vs. 27.1% in the RT group) of the overall population. The average tumor size in the chemo + RT group was 4.22 cm (SD: 2), larger than that in the RT group (mean: 3.87 cm, SD: 1.63). 50.6% of patients had an ECOG 1 performance status in the overall population, with the chemo + RT group demonstrating a higher proportion of patients with better performance status (85.2% ECOG 0–1) compared to the RT group (60.0% ECOG 0–1) (Table 3 and Table 4).

The majority of tumors were of urothelial origin (90%). Urothelial with squamous cell differentiation represented 6.5% of cases, with other rare histologies comprising the remainder. The primary reasons for opting for bladder preservation were poor health (77.1%), with patient refusal of surgery being more common in the chemo + RT group (31.1% vs. 10.6% in RT alone). Locally advanced, unresectable disease accounted for 6.9% of cases.

Maximal TURBT was performed in 74.5% of all patients, with similar rates between the RT alone (75.3%) and chemo + RT (72.1%) groups. A second TURBT was performed prior to chemoradiation in 16.5% of patients, with comparable rates in both groups (15.9% for RT alone and 18.0% for chemo + RT). NAC was administered to 34.4% of patients and adjuvant chemotherapy to 6.6% of patients, within the chemo + RT group. Concurrent chemotherapy was employed in 77.0% of patients in the chemo + RT group. Chemotherapy agents included cisplatin, carboplatin, gemcitabine, 5-FU, and mitomycin as IV infusions.

Salvage cystectomy was performed in 6.1% of patients, while 90.0% did not. The chemo + RT group had a higher proportion of salvage cystectomies (11.5%) than the RT group (4.1%). For those who underwent cystectomy, pathological T-stages ranged from T0 to T4b, with T3a and T3b being the most common.

The median follow-up was 22.8 months in the RT group (range: 1.74–173 months) and 32.7 months in the chemo + RT group (range: 3.97–150 months). 209 patients (90.5%) experienced death during the follow-up period. Disease-specific mortality was observed in 117 patients (50.6%). A total of 132 patients (57.1%) experienced disease recurrence or progression, while 99 patients (42.9%) did not (Table 5).

**Figure 1:**
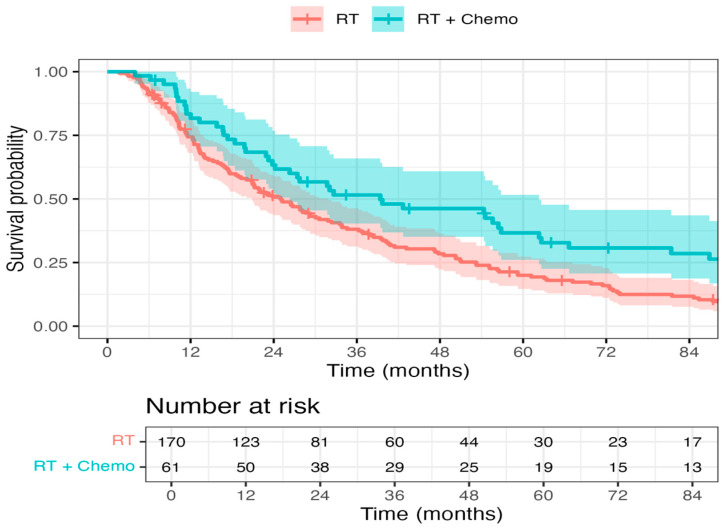
Overall survival in the RT and Chemo + RT groups: A hazard ratio of 0.70 (95% confidence interval: 0.40–1.25, *p* = 0.113) indicated a trend toward improved OS in patients receiving chemo + RT as opposed to RT alone, but this did not reach statistical significance.

**Figure 2:**
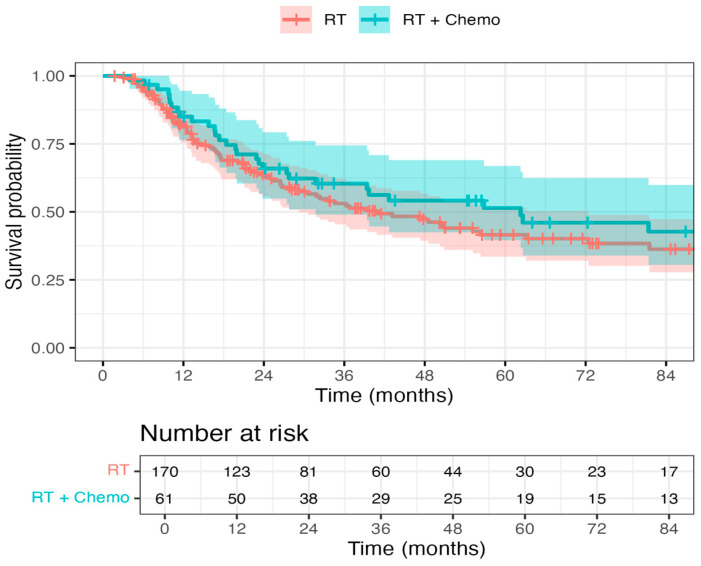
Disease-specific survival in the RT and Chemo + RT groups: The DSS endpoint demonstrated a strong trend favoring chemo + RT, with a hazard ratio of 0.63 (95% confidence interval: 0.31–1.30, *p* = 0.083).

**Figure 3:**
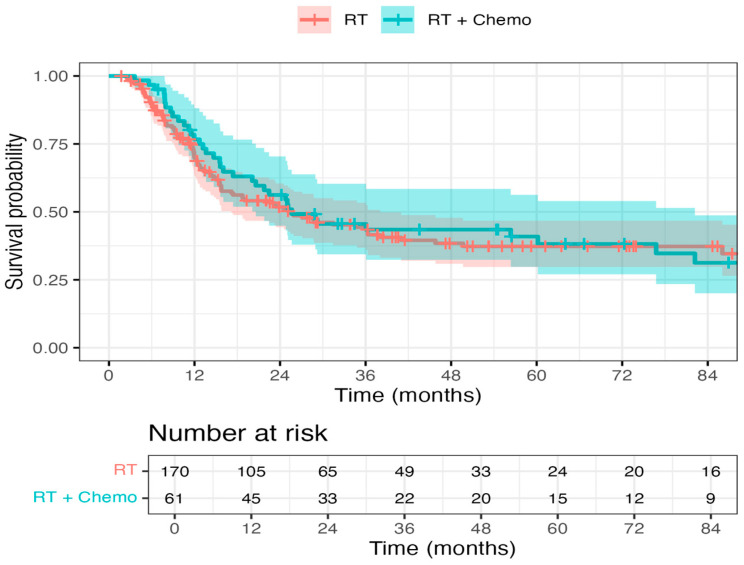
Progression-free survival in the RT and Chemo + RT groups: There was no statistically significant difference between chemo + RT and RT alone with respect to recurrence/progression (Hazard ratio of 0.80, 95% confidence interval: 0.42–1.54, *p* = 0.343).

A hazard ratio of 0.70 (95% CI: 0.40–1.25, *p* = 0.113) indicated a trend toward improved OS in patients receiving chemo + RT as opposed to RT alone, but this did not reach statistical significance (Figure 1). The DSS endpoint demonstrated a strong trend favoring chemo + RT, with a hazard ratio of 0.63 (95% CI, 0.31–1.30; *p* = 0.083) (Figure 2). There was no statistically significant difference between chemo + RT and RT alone with respect to recurrence/progression (Hazard ratio of 0.80, 95% CI: 0.42–1.54, *p* = 0.343) (Figure 3).

## 5. Discussion

The present study evaluated clinical outcomes associated with radiotherapy alone versus combined chemoradiotherapy in a real-world cohort characterized by advanced age, limited performance status, and a substantial burden of comorbidities. Our findings indicate a trend toward improved outcomes with combined chemoradiation therapy across all endpoints. However, the differences did not reach statistical significance in the chemo + RT cohort, in contrast to findings in the existing literature.

There were some notable clinical and demographic variations between the treatment groups in our patient cohort. Patients receiving RT alone were generally older (mean age, 81.2 years) than those in the chemo + RT group (mean age, 70.7 years). The age disparity likely reflects the tendency to offer more aggressive combined modality treatment to younger patients who may better tolerate the increased toxicity associated with chemotherapy. The higher proportion of T3 and T4 tumors in the chemo + RT group (44.3% vs. 27.6% in RT alone) suggests that more advanced disease may have influenced the decision to use combined therapy, which is in line with the current guidelines recommending more aggressive treatment for higher-stage tumors [6,13,14].

Despite factors typically discouraging bladder preservation, like carcinoma in situ (21.2%), tumor-associated hydronephrosis (35.5%), and multifocal tumors (27.7%), our cohort still exhibited these characteristics [15]. In line with our findings, Smith et al. reported that only around 20% of patients met the predefined criteria for TMT [16]. Beyond the factors mentioned earlier, additional factors such as patient preference for organ preservation, quality of life, comorbidities and center experience with bladder preservation strategies also influence treatment choices [17]. Nearly half of our patient cohort died from non-bladder cancer causes, highlighting the importance of comorbidities in treatment decisions, which have been shown to impact survival and limit options [18].

**Figure 4:**
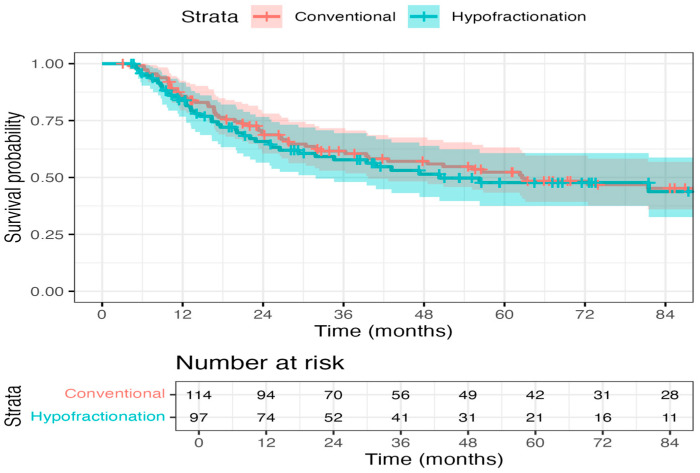
Disease-specific survival by fractionation approach: This work demonstrated overlapping DSS (*p* = 0.727) curves for hypofractionated and standard fractionation RT approaches.

Patients who received hypofractionated RT made up 42.4% of our cohort. A meta-analysis of the BC2001 and BCON trials found that a hypofractionated schedule of 55 Gy in 20 fractions achieved similar outcomes to 64 Gy in 32 fractions for invasive locoregional control and toxicity [19]. Radiation therapy with 55 Gy in 20 fractions was associated with a lower risk of locoregional invasive recurrence (adjusted HR, 0.71 [95% CI, 0.52–0.96]) with a similar toxicity profile. Thus, 55 Gy in 20 fractions is recommended as the standard of care for bladder preservation. The data presented in this work demonstrated overlapping DSS (*p* = 0.727) curves for hypofractionated and standard fractionation RT approaches (Figure 4). Several factors may explain this difference. First, the endpoints differ: the trials reported locoregional recurrence, an intermediate outcome, whereas our study evaluated DSS. Locoregional recurrence may influence DSS, but not all recurrences lead directly to bladder cancer–specific death, particularly in older or comorbid patients who may die of competing causes or undergo effective salvage therapies. Second, our study is a retrospective, real-world cohort, including a heterogeneous patient population, often older and less fit than trial participants, with variable treatment techniques and adherence. Third, the sample size may have limited statistical power to detect modest differences between fractionation schedules. Taken together, these findings suggest that while hypofractionated radiotherapy is safe and effective in clinical trials, real-world outcomes may differ due to endpoint selection, patient characteristics, and treatment heterogeneity.

Bladder preservation strategies were well tolerated in our study, as evidenced by the 90% treatment completion rate, which is comparable to rates reported in the literature. In the BC2001 trial, around 95% of patients in the chemoradiation group completed radiotherapy at the target dose [20]. This high completion rate suggests that chemoradiation therapy is generally well tolerated by patients.

The trend towards reduced disease recurrence or progression in our study (HR 0.802, *p* = 0.343) is directionally consistent with the long-term outcomes reported in the pooled analysis of RTOG bladder-preservation studies. This analysis showed 5- and 10-year estimates of muscle-invasive local failure at 13% and 14%, respectively, indicating the durability of disease control with combined-modality therapy [10]. Our study showed a 29.2% reduction in overall mortality risk (HR 0.70, *p* = 0.113) and a 36.6% reduction in bladder cancer-specific mortality (HR 0.63, *p* = 0.083) with chemo + RT versus RT alone. Although we demonstrated a trend toward reduced overall and bladder cancer-specific mortality with chemo + RT approaches compared to RT alone, that was not statistically significant. Several factors may explain this finding. First, the retrospective design and relatively small sample size limit the statistical power to detect significant differences. Additionally, the patients in the chemo + RT group were generally healthier and fitter, suggesting favorable selection bias. Furthermore, given the multicenter nature of the studies, diverse treatment patterns and referral bias may have affected our results.

A consideration in interpreting our findings is the long study period (2002–2020), during which substantial advances in radiotherapy delivery for urothelial carcinoma occurred. Early in this timeframe, bladder radiotherapy was typically delivered using 3D-conformal techniques with limited image guidance. Over the past two decades, however, practice has shifted toward more sophisticated, intensity-modulated techniques, often adaptive, combined with improved image-guidance, enabling more precise dose delivery and better sparing of normal tissues [21]. Despite these advances, only 24.7% of patients in our cohort received IMRT/VMAT, indicating that the majority were managed with earlier-generation techniques. It is possible this discrepancy may have influenced both tumor control and toxicity outcomes, potentially introducing a temporal bias. Patients treated earlier in the study period may not have benefited from the improved accuracy and conformality associated with modern radiotherapy techniques, which in contemporary practice are believed to improve bladder-preserving therapy. However, since the changes in radiotherapy techniques since 2002 were not accompanied by radiation dose-escalation in our population, they would not be expected to have had a significant impact on disease control. Rather, these improvements would be expected to have a more significant effect on treatment-related toxicity, an endpoint not investigated in the present study.

Lastly, because of the retrospective nature of this study, detailed information on acute or late toxicities related to radiotherapy or chemoradiotherapy was not consistently available and therefore could not be reliably analyzed. We acknowledge this as a limitation, particularly given that bladder-preserving strategies are often used in older or medically frail patients for whom treatment tolerability is a key consideration. The lack of toxicity data limits our ability to comment on the comparative safety profiles of the evaluated treatment approaches.

Overall, we observed a trend favoring chemo + RT over RT alone for bladder preservation in MIBC; however, the lack of statistical significance suggests that our findings do not fully align with existing evidence. It is a reminder that the statistically significant improvements observed in randomized trials involving highly selected patient cohorts are not always realized in the real-world setting of heterogeneous patient populations, as the present study highlights. This discrepancy also underscores the pivotal role of patient selection in determining treatment success, as inadequate selection or suboptimal matching of patients to treatment strategies may compromise outcomes and obscure potential therapeutic benefits.

Further biomarker-driven research is warranted to better define which patients are most likely to benefit from chemotherapy intensification, whether delivered concurrently, neoadjuvantly, or adjuvantly, and which may achieve durable control with RT alone [22,23]. Emerging tools such as circulating tumor DNA (ctDNA) and urinary tumor DNA (utDNA) hold promise for refining risk stratification and guiding individualized treatment decisions [24,25,26]. Treatment regimen selection is particularly critical given the advanced age and high prevalence of comorbidities among patients with MIBC, especially in the era when biomarker-driven treatment escalation/de-escalation strategies are considered. The evolving understanding of MIBC as a systemic disease further supports the potential of RT-based strategy as an effective alternative to surgery. Prospective, biomarker-integrated trials are essential to elucidate the biological and clinical factors that can optimize patient-tailored management and improve outcomes.

## 6. Conclusions

In summary, although existing literature consistently demonstrates superior bladder-preservation outcomes with chemoradiotherapy compared with radiotherapy alone, our study showed only a non-significant trend in this direction. This divergence from published findings suggests that differences in patient characteristics may influence the relative benefit of each approach. Our results imply that not all patients derive the same advantage from chemoradiotherapy: for some individuals, radiotherapy alone may be sufficient, whereas others may require the added intensity of chemoradiation to optimize outcomes. These findings reinforce the importance of careful, individualized patient selection when considering bladder-preserving strategies in MIBC. Advances in biomarker-driven strategies, including ctDNA and utDNA, alongside prospective, tailored trials, are essential to optimize treatment intensification, guide personalized therapy, and improve outcomes in this patient population.

## Figures and Tables

**Table 1:** Assessment of the standard mean difference (SMD) of each covariate before weighting.

Variable	RT	Chemo + RT	SMD
Age	81.24	70.67	−1.19
ECOG	1.34	0.85	−0.69
pT = T2	0.72	0.56	−0.35
pT = T3	0.18	0.28	0.23
pT = T4	0.09	0.16	0.21
CIS	0.19	0.26	0.16
Tumor-associated HN	0.34	0.41	0.15
Multifocal tumor	0.27	0.30	0.05
Tumor size (cm)	3.89	4.12	0.16
Creatinine	122.44	92.70	−0.59

ECOG: Eastern Cooperative Oncology Group, CIS: Carcinoma in situ, HN: Hydronephrosis, SMD: Standardized Mean Difference.

**Table 2:** Assessment of the standard mean difference (SMD) of each covariate after weighting.

Variable	RT	Chemo + RT	SMD
Age	76.36	76.46	0.01
ECOG	1.06	1.05	−0.01
pT = T2	0.66	0.67	0.01
pT = T3	0.26	0.26	0.00
pT = T4	0.08	0.07	−0.04
CIS	0.23	0.24	0.03
Tumor-associated HN	0.33	0.32	−0.03
Multifocal tumor	0.29	0.28	−0.02
Tumor size (cm)	3.99	4.01	0.01
Creatinine	98.75	98.44	−0.01

ECOG: Eastern Cooperative Oncology Group, CIS: Carcinoma in situ, HN: Hydronephrosis, SMD: Standardized Mean Difference.

**Table 3:** Baseline patient characteristics.

	RT (*n* = 170)	Chemo + RT (*n* = 61)	Overall (*n* = 231)	*p*-Value
Age at Diagnosis				
Mean (SD)	81.2 (7.30)	70.7 (10.2)	78.5 (9.39)	<0.001
Median [Min, Max]	83.0 [44.0, 95.0]	72.0 [48.0, 88.0]	81.0 [44.0, 95.0]	
Gender				
Female	47 (27.6%)	12 (19.7%)	59 (25.5%)	0.292
Male	123 (72.4%)	49 (80.3%)	172 (74.5%)	
pT				
T2	123 (72.4%)	34 (55.7%)	157 (68.0%)	0.056
T3	31 (18.2%)	17 (27.9%)	48 (20.8%)	
T4	16 (9.4%)	10 (16.4%)	26 (11.3%)	
CIS				
No	58 (34.1%)	20 (32.8%)	78 (33.8%)	0.515
Yes	33 (19.4%)	16 (26.2%)	49 (21.2%)	
Missing	79 (46.5%)	25 (41.0%)	104 (45.0%)	
Tumor-associated HN				
No	113 (66.5%)	36 (59.0%)	149 (64.5%)	0.375
Yes	57 (33.5%)	25 (41.0%)	82 (35.5%)	
Multifocal Tumor				
No	124 (72.9%)	43 (70.5%)	167 (72.3%)	0.842
Yes	46 (27.1%)	18 (29.5%)	64 (27.7%)	
Tumor size (cm)				
Mean (SD)	3.87 (1.63)	4.22 (2.00)	3.98 (1.76)	0.326
Median [Min, Max]	3.80 [1.30, 8.50]	4.00 [1.20, 10.0]	3.90 [1.20, 10.0]	
Missing	77 (45.3%)	19 (31.1%)	96 (41.6%)	
Creatinine (umol/L)				
Mean (SD)	125 (69.0)	92.4 (27.3)	116 (62.2)	<0.001
Median [Min, Max]	108 [35.0, 608]	88.5 [37.0, 198]	99.0 [35.0, 608]	
Missing	16 (9.4%)	3 (4.9%)	19 (8.2%)	
ECOG				
0	19 (11.2%)	18 (29.5%)	37 (16.0%)	<0.001
1	83 (48.8%)	34 (55.7%)	117 (50.6%)	
2	58 (34.1%)	9 (14.8%)	67 (29.0%)	
3	9 (5.3%)	0 (0%)	9 (3.9%)	
Missing	1 (0.6%)	0 (0%)	1 (0.4%)	
Maximal TURBT				
No	42 (24.7%)	17 (27.9%)	59 (25.5%)	0.753
Yes	128 (75.3%)	44 (72.1%)	172 (74.5%)	
Second TURBT prior to treatment				
No	143 (84.1%)	50 (82.0%)	193 (83.5%)	0.851
Yes	27 (15.9%)	11 (18.0%)	38 (16.5%)	
Neoadjuvant or Adjuvant chemotherapy				
No	170 (100%)	37 (60.7%)	207 (89.6%)	<0.001
Yes	0 (0%)	24 (39.3%)	24 (10.4%)	
Reason for bladder preservation				
Poor health	149 (87.6%)	29 (47.5%)	178 (77.1%)	<0.001
LA/unresectable	3 (1.8%)	13 (21.3%)	16 (6.9%)	
Refusal of surgery	18 (10.6%)	19 (31.1%)	37 (16.0%)	
RT technique				
3DCRT	133 (78.2%)	41 (67.2%)	174 (75.3%)	0.124
IMRT/VMAT	37 (21.8%)	20 (32.8%)	57 (24.7%)	
Salvage cystectomy				
No	157 (92.4%)	51 (83.6%)	208 (90.0%)	0.074
Yes	7 (4.1%)	7 (11.5%)	14 (6.1%)	
Missing	6 (3.5%)	3 (4.9%)	9 (3.9%)	
Recurrence site				
Distant	19 (11.2%)	20 (32.8%)	39 (16.9%)	0.069
Local	35 (20.6%)	15 (24.6%)	50 (21.6%)	
Missing	116 (68.2%)	26 (42.6%)	142 (61.5%)	
Total number of fractionations				
Hypofractionated RT	91 (53.5%)	5 (8.2%)	96 (41.6%)	<0.001
Standard fractionation	63 (37.1%)	54 (88.5%)	117 (50.6%)	
Incomplete treatment	16 (9.4%)	2 (3.3%)	18 (7.8%)	

SD: Standard deviation, CIS: Carcinoma in situ, *HN:* Hydronephrosis, Eastern Cooperative Oncology Group (ECOG) performance-status scores range from 0 to 5, with higher scores indicating greater disability, TURBT: Trans urethral resection of bladder tumor. *p*-values were derived from *T*-test for continuous variables and from a Chi-Square test for categorical variables.

**Table 4:** Radiation dose and fractionation in the overall population.

	19–24 (*n* = 96)	25+ (*n* = 117)	Incomplete Treatment (*n* = 18)	Overall (*n* = 231)
Total RT Dose				
50–55 Gy	91 (94.8%)	8 (6.8%)	2 (11.1%)	101 (43.7%)
55–60 Gy	3 (3.1%)	60 (51.3%)	0 (0%)	63 (27.3%)
>60 Gy	0 (0%)	48 (41.0%)	0 (0%)	48 (20.8%)
Missing	2 (2.1%)	1 (0.9%)	16 (88.9%)	19 (8.2%)

**Table 5:** Outcomes of the patient population: Mortality, disease-specific death, and disease recurrence or progression.

Outcomes	Overall (*n* = 231)
Mortality	
No	22 (9.5%)
Yes	209 (90.5%)
Disease-specific death	
No	114 (49.4%)
Yes	117 (50.6%)
Disease recurrence or progression	
No	99 (42.9%)
Yes	132 (57.1%)

## Data Availability

The original contributions presented in this study are included in the article. Further inquiries can be directed to the corresponding authors.
